# Genetic variants at HbF‐modifier loci moderate anemia and leukocytosis in sickle cell disease in Tanzania

**DOI:** 10.1002/ajh.23859

**Published:** 2014-10-20

**Authors:** Siana Nkya Mtatiro, Julie Makani, Bruno Mmbando, Swee Lay Thein, Stephan Menzel, Sharon E. Cox

**Affiliations:** ^1^ Muhimbili Wellcome ProgrammeMuhimbili University of Health and Allied SciencesDar‐es‐SalaamTanzania; ^2^ Department of Biological SciencesDar es Salaam University College of Education, Dar es SalaamTanzania; ^3^ Nuffield Department of MedicineUniversity of OxfordUnited Kingdom; ^4^ Division of Cancer StudiesMolecular Haematology, King's College LondonLondonUnited Kingdom; ^5^ Department of Haematological MedicineKing's College Hospital NHS Foundation TrustLondonUnited Kingdom; ^6^ Faculty of Epidemiology & Population HealthMRC International Nutrition Group, London School of Hygiene & Tropical MedicineLondonUnited Kingdom

## Abstract

Fetal hemoglobin (HbF) is a recognized modulator of sickle cell disease (SCD) severity. HbF levels are strongly influenced by genetic variants at three major genetic loci, *Xmn1‐HBG2, HMIP‐2*, and *BCL11A*, but the effect of these loci on the hematological phenotype in SCD, has so far not been investigated. In a cohort of individuals with SCD in Tanzania (HbSS and HbS/β° thalassemia, *n* = 726, aged 5 or older), HbF levels were positively correlated with hemoglobin, red blood cell (RBC) indices, mean corpuscular volume (MCV), and mean corpuscular hemoglobin (MCH), and negatively with white blood cell (WBC) and platelet counts (all *P* < 0.0001). We subsequently assessed the contribution of the three HbF modifier loci and detected diverse effects, including a reduction in anemia, leukocytosis, and thrombocytosis associated with certain HbF‐promoting alleles. The presence of the ‘T’ allele at *Xmn1‐HBG2* led to a significant increase in hemoglobin (*P* = 9.8 × 10^−3^) but no changes in cellular hemoglobin content. *Xmn1‐HBG2* ‘T’ also has a weak effect decreasing WBC (*P* = 0.06) and platelet (*P* = 0.06) counts. The *BCL11A* variant (*rs11886868*‐‘C’) increases hemoglobin (*P* = 2 × 10^−3^) and one of the *HBS1L‐MYB* variants decreases WBC values selectively (*P* = 2.3 × 10^−4^). The distinct pattern of effects of each variant suggests that both, disease alleviation through increased HbF production, and ‘pleiotropic’ effects on blood cells, are involved, affecting a variety of pathways. Am. J. Hematol. 90:E1–E4, 2015. © 2014 Wiley Periodicals, Inc.

## Introduction

Sickle cell disease (SCD) is an inherited hemoglobin disorder caused by the Glu6Val mutation in the β globin chain. It has a devastating impact in Sub‐Saharan Africa, where it is highly prevalent and a significant cause of childhood mortality 1. The severity of the disease presentation is variable, and is usually reduced in patients retaining high levels of fetal hemoglobin (HbF) into adulthood 2, a condition that is strongly influenced by secondary genetic factors. Common genetic variants promoting HbF persistence have been identified at three loci: a promoter variant of the gene encoding the ^G^γ globin chain of HbF (termed *Xmn1‐HBG2*) and clusters of variants in regulative elements for two hematopoietic transcription factors, *BCL11A* and *MYB*. BCL11A acts as a repressor of γ globin gene expression, whereas genetic variation near *MYB* (termed *HMIP‐2, HBS1L‐MYB* intergenic polymorphism, block 2) affects the levels of HbF indirectly by altering the kinetics of erythropoiesis 3. In healthy, nonanemic individuals, most HbF‐associated variants have small, but significant effects on general hematological parameters (“pleiotropic effects”). The *HMIP‐2* locus influences average volume (MCV), hemoglobin content (MCH), and number (RBC) of erythrocytes 4. To a lesser degree, *HMIP‐2* affects hemoglobin (Hb), hematocrit (HCT), mean corpuscular hemoglobin concentration (MCHC) 5, and also numbers of platelets (PLT), monocytes, and total white blood cells (WBC) 4, 5, 6, 7, 8, 9, 10. *BCL11A* variants have generally weaker pleiotropic effects, on RBC, MCV, and MCH 5, 8. Subtle effects such as these might be hard to discern in SCD, where blood parameters are expected to primarily reflect disease‐related processes, such as variable degrees of anemia, leukocytosis, and thrombocytosis 11, 12, 13, 14. HbF levels in SCD are also affected by the disease itself. They are generally increased, due to a combination of stress erythropoiesis, which releases more F cells 15, 16, 17, immature erythrocytes that contain relatively significant amounts of HbF 18, and also due to the selective survival of such cells 19, 20, 21. Nonetheless, a wide variation of HbF levels has been observed in patients with HbSS, which is mainly ascribed to the underlying genetic background of other coinherited factors.

We wanted to know how the natural variability in HbF levels and the known genetic HbF modifiers influence the hematological phenotype of SCD patients. For this purpose, we studied general blood cell parameters, peripheral HbF levels, and genotype at the three main HbF modifier loci, *BCL11A, HMIP‐2*, and *Xmn1‐HBG2*, in 726 Tanzanian SCD patients, who have minimal disease intervention such as regular blood transfusion or hydroxyurea therapy.

## Methods

The Muhimbili Sickle Cell Cohort has been described previously 1. Ethics approval is in place from the Muhimbili University Research and Publications Committee (MU/RP/AEC/VOLX1/33).

Confirmation of diagnosis (Hb SS or HbS/β^0^ thalassemia genotype) and HbF quantification were carried out by High Performance Liquid Chromatography (Variant I, Biorad, Hercules, CA). Hematological parameters (Hb, RBC, MCV, MCH, MCHC, WBC, PLT, and platelet volume‐MPV) were measured with an ABX Pentra 60 Analyzer (Horiba, Kyoto, Japan). Mean/median values for our cohort are shown in the Supporting Information Table SI.

Patients were excluded if they were on hydroxyurea therapy, younger than 60 months of age, tested malaria‐positive, had pain, fever, or had been hospitalized 30 days before or after study, or were lacking alpha thalassemia (3.7 deletion) data. Only patients with Hb SS or HbS/β^0^ thalassemia genotype were included. This resulted in a study population of 726 patients (52% females), aged 5–43 years (median 11 years, interquartile range 8–15 years).

Genetic variants were selected from ten SNPs genotyped at the three main HbF modifier loci, resulting a set of four, one effectively tagging HbF‐associated genetic variability at each locus 22, including the two sub‐loci (*A* and *B*) present at *HMIP‐2* in individuals of African descent (Table 1). They were genotyped by TaqMan procedure (Applied Biosystems, Foster City, CA): *rs11886868* (for *BCL11A*), *rs9389269* (for *HMIP‐2B*), and *rs7482144* (*Xmn1‐HBG2*, after PCR), or by PCR fragment sizing (*rs66650371*, for *HMIP‐2A*) 22, 23, 24. Alpha thalassemia (3.7 deletion) was genotyped using a PCR based method 26.

**Table 1:** SNP Markers Used to Tag the Main HbF Modifier Loci

Chromosome locus	Chr. 2 *BCL11A*	Chr. 6 *HMIP‐2A*	*HMIP‐2B*	Chr. 11 *Xmn1‐HBG2*
SNP	*rs11886868*	*rs66650371*	*rs9389269*	*rs7482144*
Position	60,720,496	135,418,633	135,427,159	5,276,419
Allele change	T→C	In→Del	T→C	C→T
N/MAF	764/0.29	727/0.03	764/0.03	723/0.01
H/W_(p (1DF)	0.29	0.85	0.80	0.96
G. success (%)	99	94.29	99	95.71

Chromosomal position is given in hg19 co‐ordinates.

The *HMIP* locus is divided into *HMIP*‐*2A* and *HMIP‐2B*, as recently proposed (34).

*rs66650371* is characterized by presence/absence of a ‘TAY’ trinucleotide.

MAF: Minor allele frequency within the patient cohort.

H/W_(p (1DF): Hardy‐Weinberg *P*‐value, i.e., all four markers are in equilibrium.

G. success (Genotyping success): percent of individuals with genotype among those tested.

Multiple linear regression (STATA v.12, Stata Corp, College Station, TX) was used to test for association of genetic markers with hematological parameters and HbF, as well as for the influence of HbF on blood cell parameters. Alpha‐thalassemia status (3.7 deletion), age (fitted as square or native value) and sex were included *a priori* in all regression models. WBC, MPV, and HbF were log‐transformed. The genetic association analysis of the four tag markers with hematological parameters was repeated with ln[HbF] as a covariate, to test for the dependence of genetic effects on HbF levels.

## Results

We detected a significant influence of HbF levels and of variants at three major HbF modifier loci, *BCL11A, HMIP*, and *Xmn1‐HBG2*, on the hematological phenotype of Tanzanian SCD patients.

### Influence of HbF levels on hematological parameters

HbF levels associated positively with hemoglobin (Hb Beta = 0.05, *P* = 5.49 × 10^−6^), and negatively with WBC (lnWBC Beta = −0.01, *P* = 4.23 × 10^−5^) and platelet counts (Beta = 7.62, *P* = 6.3 × 10^−6^). Hb gains with higher HbF were accompanied by increases in MCV (Beta = 0.43, *P* = 9.9 × 10^−9^) and MCH (Beta = 0.16, *P* = 2.95 × 10^−9^), while RBC was unchanged (*P* = 0.97).

### Influence of genetic HbF modifier variants on HbF and on hematological parameters

HbF levels were strongly influenced by all four variants tested, confirming previous findings 24 (Table 2). The number of HbF‐promoting alleles across all genotyped markers (‘Summary Score’) was positively associated with Hb, MCV, and MCH, similar to the pattern of effects exerted by HbF itself. Individual loci; however, had diverse effects (Table 2). The rare HbF‐promoting allele at *Xmn1‐HBG2* (*rs7482144*‐‘T’) had by far the largest allelic effect on Hb (Beta = 0.79, *P* = 9.8 × 10^−3^), with a tendency towards increased RBC (Beta = 0.28, *P* = 0.06), but no change in MCV and MCH values (*P* > 0.1). When adjusting for the influence of HbF levels, the Hb‐increasing effect of *rs7482144*‐‘T’ remained large and significant (Beta = 0.69, *P* = 0.03) suggesting independence of this relationship from HbF levels. *BCL11A* (*rs11886868*‐‘C’) also had a significant effect on Hb (Beta = 0.19, *P* = 2 × 10^−3^), but this ceased to be significant after adjusting for HbF levels (*P* > 0.1). The two HbF‐increasing variants at *HMIP‐2* (*rs66650371*‐‘del’, representing sub‐locus *HMIP‐2A* and *rs9389269*‐‘C’, representing *HMIP‐2B*) are uncommon in our population and no significant effect on Hb was detected. However, the *HMIP‐2B* variant had a significant positive effect on MCV and MCH and the *HMIP‐2A* variant had a negative effect on WBC (Beta = −0.19, *P* = 2.3 × 10^−4^), which was not diminished when adjusting for HbF.

**Table 2:** Regression Analysis Testing the Influence of HbF and HbF Modifier Loci on Hematological Parameters in Tanzanian Patients with Sickle Cell Disease

Outcome variables	HbF (lnHbF%)	Genetic HbF modifiers				
		*rs11886868 (BCL11A)*	*rs66650371 (HMIP‐2A)*	*rs9389269 (HMIP‐2B)*	*rs7482144 (Xmn1‐HBG2)*	Summary score^a^
lnHbF%	–	**0.37 (5.23 × 10^−21^)**	**0.51 (2.88 × 10^−5^)**	**0.44 (3.86 × 10^−5^)**	**0.55 (4.35 × 10^−3^)**	**0.40 (8.57 × 10^−31^)**
Hb	**0.05 (5.5 × 10^−6^)**	**0.19 (2 × 10^−3^)**	0.29 (0.14)	0.10 (0.54)	**0.79 (9.8 × 10^−3^)**	**0.21 (1.51 × 10^−4^)**
RBC	−0.0002 (0.97)	0.03 (0.36)	0.004 (0.96)	−0.05 (0.49)	0.28 (0.06)	0.03 (0.35)
MCV	**0.43 (9.9 × 10^−9^)**	0.72 (0.10)	1.82 (0.18)	**2.74 (0.02)**	2.15 (0.32)	**1.05 (5.5 × 10^−3^)**
MCH	**0.16 (3.0 × 10^−9^)**	0.26 (0.11)	0.74 (0.14)	**0.96 (0.02)**	0.12 (0.88)	**0.36 (0.01)**
MCHC	0.02 (0.14)	0.07 (0.44)	0.13 (0.61)	0.06 (0.78)	−0.29 (0.48)	0.05 (0.49)
lnWBC	−**0.01 (4.2 × 10^−5^)**	5.7 **×** 10^−5^ (1)	−**0.19 (2.3 ×10^−4^)**	0.02 (0.67)	−0.149 (0.06)	−0.02 (0.21)
PLT	**7.62 (6.3 × 10^−6^)**	−9.82 (0.31)	33.29 (0.27)	22.36 (0.39)	−92.19 (0.06)	−6.43 (0.45)
lnMPV	0.001 (0.57)	0.0002 (0.98)	−0.018 (0.28)	−0.0007 (0.97)	−0.009 (0.73)	−0.002 (0.73)

Shown are regression coefficient (Beta) estimates and significance (in brackets). Age, sex, and alpha globin status were included as covariates. For the genetic data, Beta serves as a measure of the effect of an allele change from low‐HbF to high‐HbF allele. Nominally significant effects are in bold font. *N* = 664–721.

a The total number of high‐HbF alleles present in a patient.

### Additive effect of Xmn1‐HBG2 and BCL11A alleles

**Figure 1:**
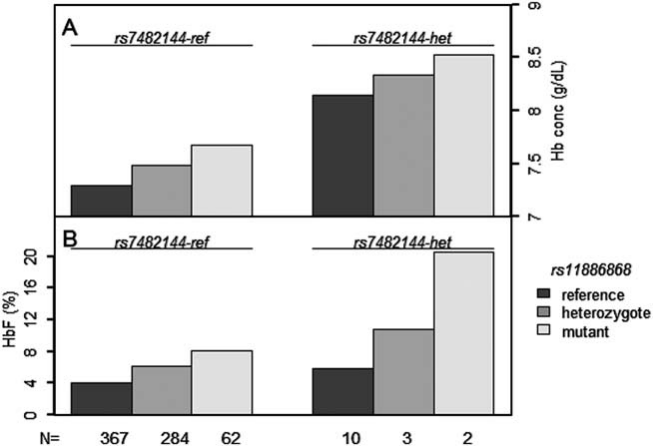
Coinheritance of *rs11886868* and *rs7482144* alleles and hemoglobin concentration. Panel (A) effect of coinheritance of *rs7482144* reference genotype (left) or heterozygous (right) with either reference, heterozygous or mutant genotypes for *rs11886868* on Hb levels. Panel B: effect of coinheritance of *rs7482144* reference genotype (left) or heterozygous (right) with either reference, heterozygous or mutant genotypes for *rs11886868* on HbF levels. There were no homozygotes for *rs7482144* high‐HbF allele. The number of individuals in each combination is presented at the bottom.

When the impact of both loci was analyzed in a joint regression model, estimates of allelic effects were similar to those obtained in separate analysis (*rs11886868*: Beta = 0.19, *P* = 0.002, *rs7482144*: Beta = 0.79, *P* = 9.8 × 10^−3^), suggesting that they contribute independently and therefore beneficial effects might add their effects when occurring in the same individual. Accordingly, patients with HbF‐promoting alleles at both loci (one at *Xmn1‐HBG2* and either one or two at *BCL11A*) had Hb levels of up to 8.5 g/dl on average, compared with 7.3 g/dl for patients lacking any such allele (Fig. 1).

## Discussion

We report that both, increased HbF levels and HbF‐promoting alleles at HbF modifier loci significantly reduce anemia, leukocytosis, and thrombocytosis in Tanzanian patients with SCD.

The beneficial effects of higher HbF on hematological parameters, such as a higher Hb, lower WBC, and platelet counts, have previously been described in Jamaica 27, 28, 29, 30, but might be less evident in a setting where the most‐severely anemic patients are transfused regularly. The reduction in anemia we saw in patients with higher HbF levels was paralleled by an increase in two RBC indices (MCV and MCH), while RBC numbers were unchanged. Possibly, this indicates the presence of a larger F‐cell fraction in such patients, a hypothesis that will be investigated in further studies. It should be noted that iron status, an independent cause of variability in red blood cell indices particularly MCV, has only been assessed in a sub‐set of the patients.

Effects of the known genetic HbF modifiers (HbF‐increasing variants at *Xmn1‐HBG2, BCL11A*, and *HMIP‐2*) were, in general, similar to that of HbF itself: Hb, MCV, and MCH were increased when the loci were analyzed jointly, using a summary score. However, individual analysis of the four variants revealed distinct patterns of effects, suggesting that diverse biological mechanisms are involved. The ‘T’ allele at *Xmn1‐HBG2* is a component of the ‘Arab‐Indian’ β globin gene cluster haplotype 31, 32, 33, known to be associated with higher HbF values and milder sickle disease. In the seven patients carrying a *Xmn1‐HBG2‐T* allele, Hb was raised, but MCV and MCH were not, probably due to the direct [34], ‘pancellular’ effect of β globin cluster variants on HbF production.

*BCL11A* (*rs11886868*‐C) had a significant effect on Hb, which was HbF‐dependent. The HbF increase due to this allele (Beta) was small, but as it is highly prevalent in this population (29% allele frequency), it created an overall significant impact. Five patients had HbF increasing alleles at both *BCL11A* and *Xmn1‐HBG2* loci, resulting in maximum Hb levels (Fig. 1). Joint regression analysis of both loci supports a model of independence of their effects on Hb levels, and an additive contribution to overall hemoglobin variability. However, this will have to be confirmed in a larger population. HbF‐promoting alleles at *HMIP* are infrequent in Tanzanian patients (frequency of <0.20) and we detected no effect on Hb. *HMIP‐2B* (*rs9389269*) does influence MCV and MCH, a finding that resembles pleiotropic *HMIP‐2* effects observed in nonanemic individuals 4, 5, 7, 8, 10. *HMIP‐2A*, but not *HMIP‐2B*, has a striking effect on WBC, independent of HbF. *HMIP‐2* variants have been reported to influence the WBC count in healthy populations 9, but a possible influence of population stratification, given the ethnic diversity of Tanzania, will be evaluated in further studies.

We believe that the significant effects of the three modifier loci on general blood traits we have shown represent a combination of both, disease amelioration through HbF modification and pleiotropic effects, and that the mechanisms underlying both phenomena are diverse and gene‐specific. To explore this further, we will increase the power provided by our cohort by recruiting more patients and by broadening the scope of biological systems tested. Inclusion of additional hematological data in future analysis is expected to account for part of the background variability, thus improving our ability to detect more subtle genetic effects.

## Acknowledgments

The authors thank the patients and staff of Muhimbili National Hospital, Muhimbili University of Health and Allied Sciences (MUHAS), Tanzania, Hematology Outpatient Unit and staff of King's College Hospital, London, and members of Professor Thein's Molecular Hematology group, King's College London. We extend our special thanks to J. Mgaya, H. Mariki of the Muhimbili Wellcome Programme, MUHAS and H. Rooks from Professor Thein's Molecular Hematology group. The sponsors of this study are nonprofit organizations that support science in general. They had no role in gathering, analyzing, or interpreting the data. Siana Nkya Mtatiro is a PhD candidate at Muhimbili University of Health and Allied Sciences and this work is submitted in partial fulfillment of the requirement for the PhD.

## Author Contributions

S.L.T., S.M., S.N.M., S.E.C., and J.M. designed the study. S. M and S.N.M. designed and performed the genotyping assays. B.M. performed the initial analysis. S.N.M., S.M., S.L.T., and S.E.C. wrote the manuscript and all authors commented on the drafts of the manuscript.

## Supporting information

Supporting Information
