# Supplementary material for: Genetic variants at HbF‐modifier loci moderate anemia and leukocytosis in sickle cell disease in Tanzania
Source: Am J Hematol. 2014 Oct 20;90(1):E1–4. doi: 10.1002/ajh.23859 (PMC4737118; doi:10.1002/ajh.23859)
Supplement: Supplementary file 1 — Supporting Information [file AJH-90-E1-s001.docx]

**Table S1: Summary of the steady state hematological parameters in the Tanzanian SCD cohort**

| **Hematological trait (units)** | **(HbSS)**  **n=726** |
| --- | --- |
| Hb (g/dl) | 7.4 ±1.2 |
| RBC (x 10¹²/L) | 2.8±0.6 |
| MCV (f/L) | 81.2 ± 8.6 |
| MCH (pg) | 26.7 ± 3.3 |
| MCHC (g/dl) | 32.8 ± 1.7 |
| PLT (x10⁹/L) | 458 ± 172.9 |
| HbF (%) | 4.35(2.4 -7.1)* |
| MPV (fL) | 8.3 (7.8 - 8.85)* |
| WBC (x 10⁹/L) | 13.8 (11.1-17.1)* |

Values are mean +/- SD, except for non-normally distributed traits (indicated by ‘*’), where median and interquartile range are given. Hb: total hemoglobin, RBC: red blood cell count, MCV: mean cell volume, MCH: mean cell hemoglobin, MCHC: mean cell hemoglobin concentration, PLT: platelet count, HbF: fetal hemoglobin, MPV: mean platelet volume, WBC: white blood cell count.
